# Trends in primary percutaneous coronary intervention for the treatment of acute coronary ST-elevation myocardial infarction in Latin American countries: insights from the CECI consortium

**DOI:** 10.3389/fcvm.2024.1275907

**Published:** 2024-05-17

**Authors:** Alfredo Matías Rodriguez-Granillo, Leonardo Solórzano, Gilberto Vladimir Pérez-Omaña, Diego Ascarrunz, Hernán Pavlovsky, Reynaldo Gomez-Valerio, Ignacio Bertrán, Federico Flores, Julio Parra, Juan Guiroy, Juan Mieres, Francisco Carvajal, Carlos Fernández-Pereira, Alfredo E. Rodriguez

**Affiliations:** ^1^ Interventional Cardiology Department, Centro de Estudios en Cardiología Intervencionista (CECI), Ciudad de Buenos Aires, Argentina; ^2^ Interventional Cardiology Department, Sanatorio Las Lomas, San Isidro, Provincia de Buenos Aires, Argentina; ^3^ Interventional Cardiology Department, CardioCentro, Manta, Manabí, Ecuador; ^4^ Interventional Cardiology Department, Policlínica Táchira, San Cristóbal, Táchira, Venezuela; ^5^ Interventional Cardiology Department, Clínica IMA, Adrogué, Provincia de Buenos Aires, Argentina; ^6^ Interventional Cardiology Department, Centro de Intervenciones Cardiovasculares, Santo Domingo, Dominican Republic; ^7^ Interventional Cardiology Department, Sanatorio Otamendi, Ciudad de Buenos Aires, Argentina; ^8^ Interventional Cardiology Department, InCorazón, Quito, Ecuador; ^9^ Interventional Cardiology Department, Instituto Cardiovascular del Chaco, Resistencia, Provincia de Chaco, Argentina

**Keywords:** STEMI, primary PCI, gender differences, Latin America, Caribbean, elderly

## Abstract

**Background:**

ST-elevation myocardial infarction (STEMI) requires revascularization treatment, preferably via primary percutaneous coronary interventions (pPCI). There is a lack of data about contemporary management of STEMI in Latin America.

**Methods:**

This was a multicenter, multinational, prospective, and dynamic registry of patients undergoing pPCI in Latin America for STEMI (STEMI/LATAMI Registry) that was carried out in nine centers from five countries (Argentina, Ecuador, Venezuela, Bolivia, and the Dominican Republic) between June 2021 and June 2023. All interventionalists involved in the study were originally trained at the same institution (Centro de Estudios en Cardiología Intervencionista, Buenos Aires, Argentina). The primary objective was to evaluate procedural and in-hospital outcomes of pPCI in STEMI and in-hospital outcome in the Latin America (LATAM) region; as secondary endpoints, we analyzed the following subgroups: differences between pPCI vs. pharmaco-invasive or late presenters, gender, elderly and very elderly patients, cardiogenic shock outcomes, and causes of STEMI.

**Results:**

In total, 744 STEMI patients who underwent PCI between June 2021 and June 2023 in five countries (nine centers) in our continent were included; 76.3% had a pPCI, 8.1% pharmaco-invasive PCI, and 15.6% had late STEMI PCI. There were no differences in region or center when we evaluated in-hospital and 30 days of death. The rate of procedural success was 96.2%, and the overall in-hospital mortality rate was 2.2%. In the subgroup of pPCI, mean symptom onset-to-balloon time was 295.3 ± 246 min, and mean door-to-balloon time was 55.8 ± 49.9 min. The femoral approach was chosen in 60.5%. In 3.0% of patients, the left main disease was the culprit artery, with 1.63 ± 1.00 stents per patient (564 drug-eluting stents and 652 bare metal stents), with 34 patients receiving only plain optimal balloon angioplasty. Definitive stent thrombosis was related to the infarct artery as the primary cause of STEMI in 7.5% of patients. The use of assistant mechanical devices was low, at 2.1% in the pPCI group. Women were older, with large numbers in very elderly age (≥90 years), greater mortality, and incidence of spontaneous coronary dissection as a cause of STEMI (*p* < 0.001, *p* < 0.001, *p* < 0.001, and *p* < 0.003, respectively).

**Conclusion:**

In suitable LATAM Centers from low/medium-income countries, this prospective registry in patients with STEMI, PCI performed by well-trained operators has comparable results to those reported in well-developed countries.

## 1 Introduction

Myocardial infarction is a manifestation of ischemic heart disease and constitutes a life-threatening emergency that necessitates prompt treatment, which significantly impacts the patient's prognosis. The management of this pathology follows the current classification and has shown improvement over recent decades (1). ST-elevation myocardial infarction (STEMI) denotes the thrombotic occlusion of an epicardial artery, with the current preferred strategy involving revascularization within the first 12 h of symptom onset, either through fibrinolytic agents or percutaneous coronary intervention (PCI), the latter being preferable in most scenarios (1, 2).

In its agenda for 2018–2030, the Pan-American Health Organization (PAHO) established the goal of reducing the burden of cardiovascular disease in the region. Consequently, it is imperative to assess whether the contemporary management of STEMI in Latin America is comparable to that in high-income countries (3).

In this prospective registry, we evaluated the baseline clinical and procedural characteristics, as well as in-hospital clinical outcomes of STEMI patients treated with PCI across various countries within our continent. Notably, these patients were managed by a group of interventionists trained using the same methodology.

## 2 Materials and methods

This was a multicenter, multinational prospective registry of patients undergoing primary percutaneous coronary intervention (pPCI) for acute myocardial infarction (AMI) in Latin America (LATAM), known as the Latin American Acute Myocardial Infarction (LATAMI) Registry. It was conducted in nine centers across five countries (Argentina, Ecuador, Venezuela, Bolivia, and the Dominican Republic) between June 2021 and June 2023. The registry embraced an all-comers approach, including all adult patients (age >18 years) who underwent pPCI, regardless of procedure success or the utilization of pharmaco-invasive or rescue PCI strategies.

The primary objective was to evaluate procedural and in-hospital outcomes, defined as a composite endpoint encompassing overall mortality, acute kidney injury, stent thrombosis, or emergent revascularization, associated with pPCI conducted in Latin American centers. Secondary endpoints involved the analysis of individual components of the primary endpoint and bleeding complications according with Academic Bleeding Consortium definitions, along with comparisons among various subgroups, including differences between pPCI vs. pharmaco-invasive or late presenters, gender disparities, outcomes in elderly and very elderly patients, outcomes in patients experiencing cardiogenic shock, and stent thrombosis as a cause of STEMI (4). In addition, in-hospital trends in complete or incomplete revascularization among patients with multivessel disease were assessed. Elderly patients were defined as those aged >75 years, while very elderly patients were those aged >90 years.

pPCI was defined as an emergent PCI to the infarct-related artery in the setting of STEMI, without prior fibrinolytic treatment, and performed within 12 h of symptom onset. The pharmaco-invasive PCI strategy was described as thrombolytic therapy combined with rescue PCI (in cases of failed thrombolysis) or systematic PCI within 2–24 h after thrombolysis.. Late PCI referred to intervention in patients with an “evolved” STEMI, presenting 12–48 h after symptom onset. Stent thrombosis was categorized as definitive or probable. Acute kidney injury was defined as an elevation of 1.5–1.9 times the baseline creatinine levels (stage 1), 2–2.9 (stage 2), or >3 (stage 3), while emergent revascularization indicated the necessity of new unplanned revascularization during hospitalization.

**Figure 1:**
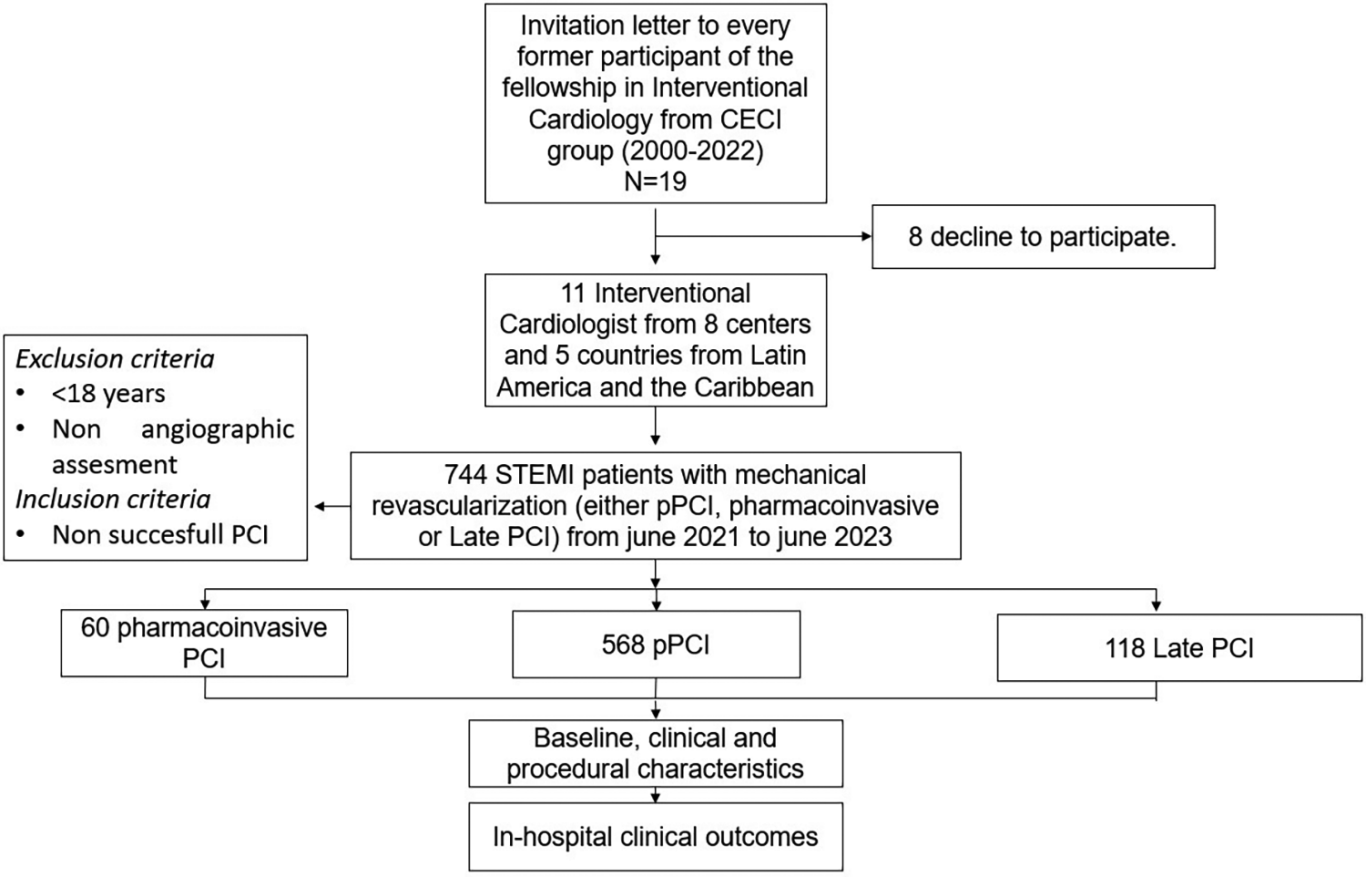
Study design. pPCI was defined as an emergent PCI to the infarct-related artery in the setting of STEMI without previous fibrinolytic treatment and within 12 h from onset symptoms. Pharmaco-invasive PCI was defined as thrombolytic therapy combined with rescue PCI (in failed thrombolysis) or systematic PCI within the first 2–24 h after thrombolysis. Late PCI was defined as the intervention in patients with an “evolved” STEMI-presenting12–48 h after symptoms initiation.

All interventionalists involved in the study were initially trained at the same institution (Centro de Estudios en Cardiología Intervencionista, CECI, Buenos Aires, Argentina) according to the norms and methodology of the Interventional Cardiologist Argentinian College (CACI, Colegio Argentino de Cardiología Intervencionista). The CECI group, established in 1992, comprises three Argentinian centers: Sanatorio Otamendi in Buenos Aires City; Sanatorio Las Lomas in San Isidro, Buenos Aires province; and Clinica IMA in Adrogué, Buenos Aires province. Since 2000, the CECI group has trained two interventional cardiologists every 3 years, all of whom were invited to participate in the registry, of which nine were ultimately included, as seen in Figure 1.

Data were collected from individual databases in each center, and patient information (blinded) was entered into a general online Google form with a unique ID for each patient and investigator. The registry protocol was presented to local authorities, approved by the Ethics Committee of the Centro de Estudios en Cardiología Intervencionista, and conducted in accordance with the Declaration of Helsinki. Patient informed consent adhered to local regulations.

Collected data encompassed demographic, clinical, angiographic, and procedural characteristics. In-hospital adverse events were also documented, with each site investigator accountable for data accuracy. All patients diagnosed with ST-segment elevation myocardial infarction who arrived at the centers and underwent pPCI were included.

Continuous variables were presented as means ± standard deviation (SD) or median [interquartile ranges (IQRs)], and categorical variables as percentages. Subgroup analysis involved age (elderly >75 years and very elderly >90 years), gender, multivessel disease, cardiogenic shock, and complete revascularization. Student *t*-tests and chi-square tests were employed to compare differences among categorical variables, while ANOVA was used for continuous variables. A two-sided *p*-value <0.05 was considered significant for all analyses. Statistical analyses were conducted using SPSS Statistics 27.0 software (IBM Corp., Armonk, NY, USA).

## 3 Results

### 3.1 Patient characteristics and clinical outcomes

In total, 744 STEMI patients who underwent PCI between June 2021 and June 2023 in five countries (nine centers) in our continent were finally included; 76.3% underwent pPCI, 8.1% underwent pharmaco-invasive PCI, and 15.6% underwent late PCI. The mean age was 63.4 ± 13.3 years, with women comprising 23.1% of the cohort. Among the entire cohort, 18.8% had a history of coronary artery disease (CAD), and 14% had experienced a previous myocardial infarction. In the pPCI subgroup, the mean symptom onset-to-balloon time was 295.3 ± 246 min, while the mean door-to-balloon time was 55.8 ± 49.9 min. The femoral approach was chosen in 60.5% of cases, including 2.7% of the initial radial approach group converted to femoral. Left main coronary artery (LMCA) disease was identified as the culprit artery in 3.0% of patients, with a mean of 1.63 ± 1.00 stents per patient [564 drug-eluting stents (DES) and 652 bare metal stents (BMS)]. In addition, 34 patients received plain optimal balloon angioplasty (POBA). Definitive stent thrombosis was attributed to the infarct artery as the primary cause of STEMI in 7.5% of patients. Multiple vessel disease was observed in 51.6% of patients, with 82.8% of them achieving complete revascularization either during the same procedure or in stages. Clopidogrel was the selected P2Y12 inhibitor in 53.5% of cases, and intracoronary IIb/IIIa inhibitors were administered in 55.4% of interventions. The comprehensive list of baseline demographic and clinical characteristics of the overall population is presented in Table 1. Clinical outcomes are detailed in Table 2, with an overall death rate of 2.2%. Procedural characteristics are outlined in Table 3.

**Table 1:** Demographic, clinical, and procedural characteristics of the overall population (*n* = 744 patients).

Demographic and clinical characteristics	
Age (years)	63.3 ± 13.3
Symptom onset-to-balloon time (min)	295.3 ± 246
Door-to-balloon time (min)	55.8 ± 49
Male gender (%)	76.9
High blood pressure (%)	58.3
Dyslipidemia (%)	44.9
Current smoker (%)	23.7
Diabetes (%)	23.4
Family history of CAD (%)	10.5
Known CAD (%)	18.8
Previous myocardial infarction (%)	14.0
Previous revascularization procedure (%)	13.7
Previous PCI (%)	12.6
Previous CABG (%)	1.9
Procedural characteristics	
Primary PCI (%)	76.3
Pharmaco-invasive PCI (%)	8.1
Late PCI (%)	15.6

; CABG, coronary artery by-pass surgery.

**Table 2:** Procedural and in-hospital outcomes (*n* = 744 patients).

Overall death (%)	2.2
Stent thrombosis (%)	2.6
Acute kidney injury (%)	2.4
Emergent revascularization (%)	2.6
Composite ischemic primary endpoint (%)	6.9

**Table 3:** Procedural characteristics of the overall population (*n* = 744 patients).

Procedural characteristics	
Primary PCI %)	76.3
Pharmaco-invasive PCI (%)	8.1
Late PCI (%)	15.6
Femoral access (%)	60.5
Infarct-related artery	
Left main (%)	3.0
Left arterial descendent artery (%)	50.5
Saphenous vein graft (%)	0.5
Multiple vessel disease (%)	51.6
Spontaneous coronary artery dissection (%)	0.5
In-hospital complete revascularization (%)	49.5
Programmed complete revascularization (%)	82.5
In-stent thrombosis (%)	7.5
Patients with DES implantation (%)	44.6
Patients with BMS implantation (%)	47.3
Patients with both DES and BMS implantation (%)	3.5
POBA (%)	4.6
N stent per patient	1.63 ± 1.0
Clopidogrel (%)	53.5
TIMI thrombus grade 0 or I (%)	77.9
No reflow (%)	7.8
Intracoronary IIb/IIIa inhibitors (%)	55.4
Manual thrombectomy (%)	18.3
Contrast (ml)	188 ± 65
Procedural success (%)	96.2

### 3.2 Subgroup analyses

**Figure 2:**
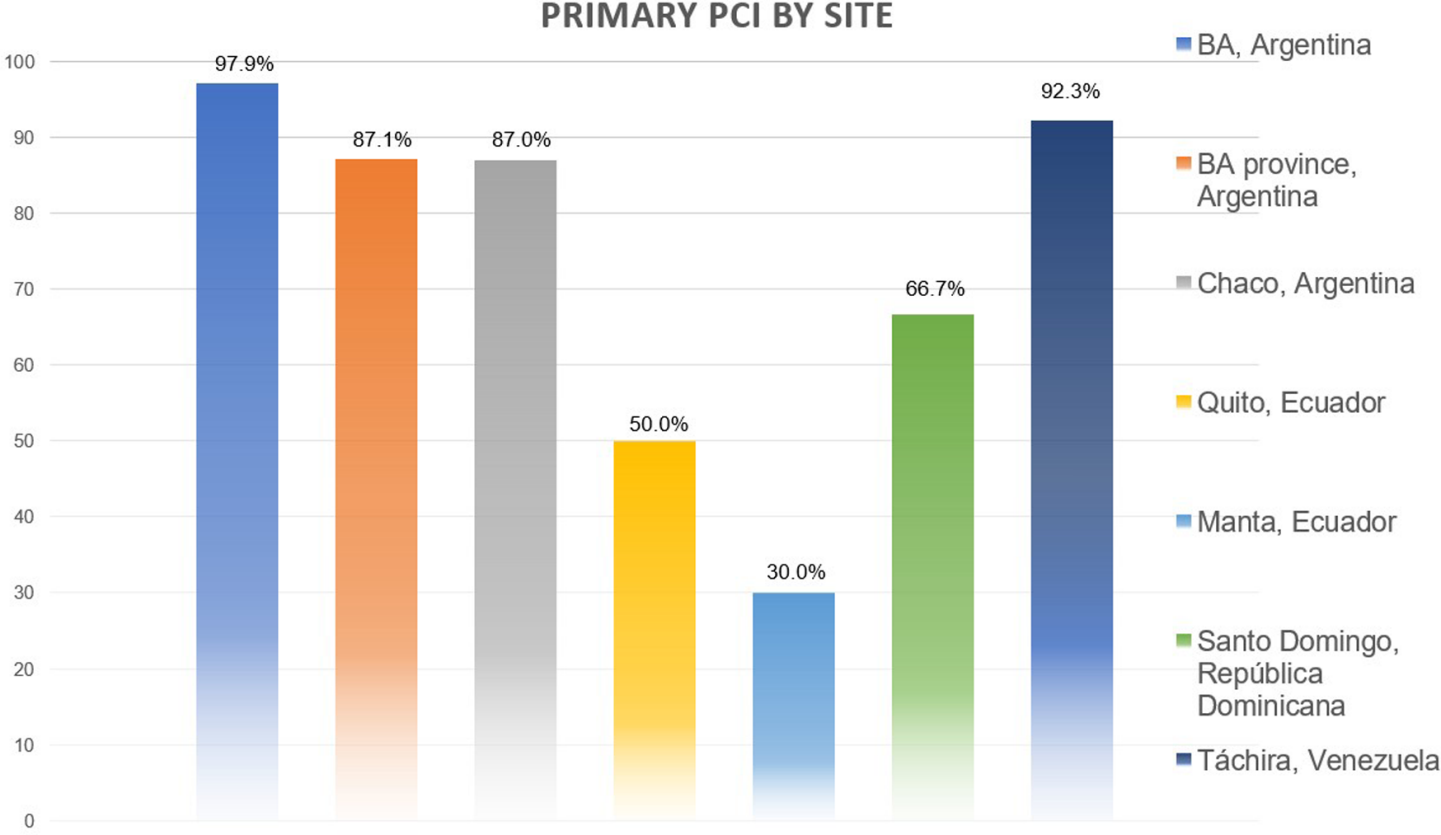
Differences in primary PCI access between regions.

We did not observe any regional or center-based disparities when assessing in-hospital and 30-day mortality. However, discrepancies were noted concerning the type of PCI (primary, pharmaco-invasive, and late), with a higher prevalence of the latter observed in less populated areas, as illustrated in Figure 2.

#### 3.2.1 Primary PCI

In the subgroup analyses, there were no significant age differences between patients who underwent pPCI and those who did not, with mean ages of 64.5 ± 13.4 and 62.8 ± 12.5 years, respectively (*p* = 0.12). However, there was a trend toward a higher proportion of primary interventions in men compared to women (78.0% vs. 70.9%, *p* = 0.057). Of note, there was a significant difference between non-diabetics (78.9%) and diabetics (67.8%) in terms of pPCI utilization (*p* = 0.002). Although there were no significant differences observed in previous history of myocardial infarction (*p* = 0.17), a significantly higher number of patients underwent revascularization in the pPCI group compared to late presenters (87.2% vs. 12.8%, *p* = 0.004).

Newer 2PY12 inhibitors (prasugrel and ticagrelor) were preferred over clopidogrel in the primary PCI group (54.1% vs. 19.3%, *p* < 0.001), and the femoral approach was the predominant access route (69.4% vs. 31.8%, *p* < 0.001).

In the pPCI group, LMCA disease was identified as the culprit artery in a higher proportion compared to non-primary PCI procedures (3.5% vs. 1.1%, *p* = 0.07). In addition, a greater number of stents were deployed in non-primary PCI procedures (1.82 ± 1.1 vs. 1.57 ± 0.94, *p* = 0.03), and although there were no differences in terms of TIMI 0–1 flow nor no-reflow phenomena in the infarct artery (*p* = 0.43 and *p* = 0.11, respectively), the use of thrombus-aspiration devices was significantly higher in the pPCI group (20.6% vs. 11.4%, *p* = 0.006) as was the intracoronary administration of IIb/IIIa inhibitors (13.2 ± 7 vs. 10.0 ± 2.3 ml, *p* < 0.001).

Contrast use was statistically higher in the pPCI subgroup (194 ± 64 vs. 173 ± 67 ml, *p* < 0.001). Although no significant differences were observed in overall death (*p* = 0.28) or cardiogenic shock (*p* = 0.53), the utilization of an intra-aortic balloon pump (IABP) was significantly higher in favor of pPCI (2.1% vs. 0.0%, *p* = 0.03) as was the use of a transient pacemaker (4.6% vs. 1.1%, *p* = 0.02). No intravascular imaging was used during the initial procedure.

#### 3.2.2 Gender differences

In comparing men and women, women were older, with mean ages of 71.7 ± 14.3 vs. 62.6 ± 12.5 years for men (*p* < 0.001). Similarly, the prevalence of high blood pressure was significantly higher in women compared to men (75.4% vs. 50.7%, *p* < 0.001). Men exhibited a higher incidence of coronary artery disease history (22.0% vs. 8.2%, *p* < 0.01), with a trend toward a higher incidence of previous AMI (14.3% vs. 8.2%, *p* = 0.074). As expected, men had a higher prevalence of previous revascularization (16.1% vs. 8.2%, *p* = 0.027), while women had a higher incidence of previous stroke (2.2% vs. 6.6%, *p* = 0.01).

The femoral approach was more commonly used in women compared to men (77.0% vs. 67.3%, *p* = 0.03), although there was no significant difference in the conversion rate from radial to femoral (*p* = 0.5). Women had a higher prevalence of LMCA disease (6.6% vs. 2.7%, *p* = 0.04) and spontaneous coronary artery dissection (2.3% vs. 0.0%, *p* = 0.003). Definitive stent thrombosis as the cause of STEMI was more prevalent in men compared to women (9.0% vs. 3.3%, *p* = 0.023), while clopidogrel was more commonly prescribed for women (54.1% vs. 42.6%, *p* = 0.24). However, the administration of IIb/IIIa inhibitors tended to be higher in men (50.0% vs. 59.5%, *p* = 0.06), although neither TIMI 0–1 flow (*p* = 0.36) nor non-reflow phenomena (*p* = 0.81) differed significantly between the two groups.

Overall, in-hospital mortality was significantly higher in women compared to men (6.6% vs. 1.3%, *p* = 0.001), with a numerically higher incidence of cardiogenic shock in women (8.2% vs. 4.9%, *p* = 0.12), although no IABP was utilized in the women’s group (0.0% vs. 2.7%, *p* = 0.053).

Notably, men presented with multiple vessel disease more frequently than women (53.4% vs. 39.3%, *p* = 0.006), and a higher proportion of men underwent complete revascularization during baseline hospitalization compared to women (31.1% vs. 16.7%, *p* = 0.044).

#### 3.2.3 Age

In terms of the elderly population, consisting of 170 patients aged >75 years, and the very elderly population, comprising 56 patients aged >90 years, compared with the rest of the cohort, notable differences were observed.

In the first subgroup, the mean age was 81.9 ± 5.9 years, significantly higher than that of the rest of the population (58.8 ± 9.7 years, *p* < 0.001). Younger patients demonstrated shorter symptom onset-to-balloon times compared to the elderly (median 276 vs. 356 min, *p* = 0.003), although there were no disparities in door-to-balloon times (*p* = 0.50). A higher proportion of women was observed in the elderly subgroup (*p* < 0.001), along with a higher prevalence of known coronary artery disease (27.1% vs. 16.6%, *p* = 0.002) and stroke (9.4% vs. 0.7%, *p* < 0.001). The femoral approach was more frequently employed in the elderly group (74.1% vs. 56.2%, *p* < 0.001), and there was a higher incidence of multiple vessel disease (58.8% vs. 50.2%, *p* = 0.048), although there were no differences in complete revascularization during index hospitalization (*p* = 0.45). Clopidogrel was the preferred choice in the elderly subgroup (75.3% vs. 47.3%, *p* < 0.001), with no significant differences in overall mortality (2.4% vs. 2.1%, *p* = 0.85).

In the very elderly subgroup, the mean age was 89.1 ± 3.9 years, significantly higher than the rest of the cohort (62 ± 11.5 years, *p* < 0.001). This group exhibited a numerical trend toward worse symptom onset-to-balloon times (353 ± 196 vs. 289 ± 250 min, *p* = 0.13) and significantly worse door-to-balloon times (median 78.3 vs. 54 min, *p* = 0.004). A higher proportion of women were also noted in this group (60.7% vs. 39.3%, *p* < 0.001). Notably, none of the very elderly subgroup were smokers (*p* < 0.001). The femoral approach was more frequently utilized in the very elderly group (82.1% vs. 58.5%, *p* < 0.001), with a higher incidence of left main coronary artery involvement as the infarct artery (7.1% vs. 2.6%, *p* = 0.058). The administration of IIb/IIIa inhibitors was more common in the non-elderly group (56.8% vs. 39.3%, *p* = 0.01), with no significant differences in non-reflow phenomena between the groups.

#### 3.2.4 Other subgroup's clinical outcomes

We assessed in-hospital overall mortality as the primary clinical outcome in the 44 patients who developed cardiogenic shock, revealing a significantly higher incidence of death in this subgroup compared to the general population (22.7% vs. 0.9%, *p* < 0.001). The presence of LMCA involvement as the infarct artery (18.2% vs. 2.0%, *p* < 0.001) and TIMI 0–1 flow (95.5% vs. 78.9%, *p* = 0.003) were also associated with this cohort of patients, whereas there were no significant associations found with stent thrombosis (*p* = 0.33), multiple vessel disease (*p* = 0.39), or age (62.6 ± 12.9 vs. 62.2 ± 13.2 years, *p* = 0.45). Trends were observed regarding gender, with 31.8% of women in the cardiogenic shock group (*p* = 0.11), and the incidence of no-reflow phenomenon (13.6% vs. 7.4%, *p* = 0.11).

Subgroup outcomes are presented in Supplementary Table S1 and Figure S3.

#### 3.2.5 Bleeding complications

We did not encounter any major bleeding events in our series, and overall bleeding rate was 6.3% for any type of bleeding. Detailed in-hospital and periprocedural bleeding complications according to the Bleeding Academic Research Consortium (BARC) classification are provided in Supplementary Table S2 (4).

## 4 Discussion

To the best of our knowledge, this is the first prospective registry in Latin America assessing the clinical outcomes of ST-elevation myocardial infarction treated with PCI performed by interventionalists trained at the same center.

The Centro de Estudios en Cardiología Intervencionista was established over 20 years ago to promote medical education and academic activities. Since then, more than 25 interventional cardiologists from Argentina and Latin America have completed the fellowship program at our institution. This fellowship is part of the CACI interventional cardiology program, affiliated with the Buenos Aires University. To the best of our knowledge, this is the first report to consider the impact of interventionists’ training location on patient outcomes.

According to a review by Alves et al., in-hospital mortality rates for STEMI patients in Latin America have varied widely across studies, ranging from 4.9% to 17.5%. However, there is a lack of data regarding the number of patients treated with primary PCI who died within the first 30 days (5). Similarly, in Europe, overall mortality rates have shown significant regional variation, ranging from 4.9% to 10.8%, with limited information available on the success of pPCI as a reperfusion therapy (6). In our registry, the in-hospital mortality rate was 2.2%, likely influenced by a shorter door-to-balloon time (55.8 ± 49.9 min), with 90.5% of patients receiving pPCI within 90 min. This contrasts with findings from a multicenter registry in Argentina, where only 30% of STEMI patients received pPCI within 90 min, resulting in a higher in-hospital mortality rate of 7.7%. However, our results align closely with those of the nationwide registry from Norway (NORMI), which reported an in-hospital mortality rate of 3% (7, 8).

The ISAC-STEMI COVID-19 Registry (The International Study on Acute Coronary Syndromes–ST-elevation myocardial infarction), a multinational collaboration from high-volume centers during the COVID-19 pandemic, provided insights into the treatment and outcomes of STEMI patients, reporting a median door-to-balloon time of 40 min (with 70% within 60 min) and an in-hospital mortality rate of 3.9% in 2019 (7–9). Similar findings were observed in a contemporary meta-analysis, where pPCI demonstrated an in-hospital mortality rate of 4.8% (9). During the COVID-19 pandemic, we did not analyze outcomes based on COVID-19 positivity in STEMI patients or investigate differences related to night shifts.

Since the COMPLETE Trial (Complete vs. Culprit-Only Revascularization Strategies to Treat Multivessel Disease after Early PCI for STEMI), current guidelines recommend complete revascularization as the standard treatment for patients with STEMI and multivessel disease, excluding those with cardiogenic shock (1–12). In our trial, we report a complete revascularization rate of 82.8%.

There are relevant data in the subgroup analysis, and despite being hypothesis generators, it is important to highlight them since there is a lack of contemporary data from the region (7, 13, 14).

The subgroup analysis provides relevant data, and despite being hypothesis generators, it is important to highlight them due to the lack of contemporary data from the region (7, 13, 14).

When we analyzed the subgroup aged >75 years, we found significant differences in symptom onset-to-balloon time (*p* = 0.003) compared to younger patients, while there were no differences when we compared door-to-balloon times (*p* = 0.50). This underscores the need for better communication and awareness with this population, taking into account that this subgroup had a larger history of coronary artery disease (*p* = 0.002), cerebrovascular accident (*p* < 0.001), and multiple vessel disease (*p* = 0.048), indicating previous contact with the healthcare system and secondary prevention. Even though this subgroup had a higher risk profile, there were no differences in complete revascularization during index hospitalization or in-hospital mortality compared to the younger cohort (15–17).

In the cohort of patients aged >90 years, there were worse door-to-balloon times, and they had more complex lesion morphology, with more LMCA involvement (*p* < 0.001). However, cardiogenic shock and in-hospital mortality did not differ, highlighting the importance of pPCI in this subgroup (18).

The rate of in-stent thrombosis as a cause of STEMI was 7.5% in our cohort, slightly higher than the incidence of 5.1% found in elderly patients in the worldwide ISAC-STEMI COVID-19 Registry (9). Given that none of them were due to an acute event, the lack of intracoronary imaging in our series did not allow us to assess the pathophysiology of stent thrombosis. Nonetheless, the occurrence of stent thrombosis as a cause of STEMI at rates of 5%–7.5% appears higher than expected with current stent designs and warrants further analysis. Although direct assessment of antithrombotic therapy in patients requiring oral anticoagulation for atrial fibrillation was not conducted, clopidogrel emerged as the most commonly used antiplatelet agent (*p* < 0.001). This preference may be justified by the higher costs associated with new antiplatelet agents in the Latin American region.

The number of BMS implanted in the registry is higher than in other series, which could be attributed to economic restrictions in certain regions of Latin America. However, despite these differences, there is no evidence of disparities in overall death or myocardial infarction between BMS and DES, as indicated by contemporary meta-analyses. Our findings align with this trend, showing no significant differences among stent selections (*p* = 0.42) (10–12). The intracoronary use of IIb/IIIa inhibitors was prevalent in the overall population (55.4%), with an average bolus dose of 12 ± 6 ml per patient, administered in 61% of the 580 patients with TIMI thrombus grade 0 or 1. Notably, we did not observe higher mortality in this subgroup (2.0%), which is consistent with previously published evidence from our group and other researchers (19–21). When we analyzed women, we observed similar differences to those reported in previous registries. Women tended to be older and have higher blood pressure, and clopidogrel was the preferred P2Y12 inhibitor (22, 23). As expected, spontaneous coronary artery dissection was more common in women, consistent with current evidence (24). Although there were no differences observed in TIMI flow or no-reflow, women received fewer IIb/IIIa inhibitors, and in-hospital mortality was higher (*p* = 0.001). The main findings from the gender comparisons are presented in Supplementary Table S1. In summary, there remains a treatment gap for STEMI in women, highlighting the need for dedicated programs and protocols to address these gender differences (25). Unlike other registries, all participating centers had a high volume of procedures (range of 840–1354). Therefore, analyses were not conducted to assess the relationship between operator volume and procedure-related mortality (26).

The rate of 30-day mortality in patients with cardiogenic shock is lower (23%) than that reported in previously major randomized clinical trials but similar to those in current national registries (43% and 28%, respectively) (27, 28). The small sample size, similar trained skills among operators with the PCI policy of culprit artery treatment only in cardiogenic shock, and short follow-up period could explain these findings.

Although previous studies have reported an association between coronary perforations and the incidence of the no-reflow phenomenon, we did not observe such an association in our study (29). Despite the high rate of femoral approach in our patient cohort, major bleeding events were not observed, with only 1% of patients experiencing bleeding requiring intervention, and an overall bleeding rate of 6.3%.

In the ORPKI Polish National Registry, Siudak et al. reported a decrease in mortality and bleeding with the radial approach (30). However, in our registry, the incidence of bleeding was not elevated, potentially due to operator preferences. Previously, our group published results from a patient series showing a numerical but not statistically significant difference in minor vascular events favoring the radial approach, including pseudoaneurysms managed conservatively (31).

## 5 Limitations

We identify limitations in our registry. First, these findings cannot be generalized to all centers in Latin America, although results among the participating centers were similar.

Second, the low utilization of mechanical assistant devices, such as IABP and Impella, compared to registries in more developed regions may reflect inequities and the burden of poverty in certain areas of the region; however, in-hospital mortality was comparable to other series. Nevertheless, the efficacy of these technologies during pPCI is controversial, potentially adding cost without clear benefit (28, 32).

Finally, we did not conduct a multivariate analysis to avoid drawing conclusions from a cross-sectional registry with numerous unmeasured biases. Instead, comparisons were made to identify potential caveats.

## 6 Conclusion

Our study has demonstrated that the outcomes of PCI for patients with STEMI in developing countries, when performed by well-trained operators, are comparable to those observed in developed countries with higher resources. This finding underscores the pivotal role of specialized training in bridging the gap in cardiac care quality between developed and developing nations. It proves the necessity for well-structured programs dedicated to training more interventional cardiologists. Such initiatives are essential for maintaining the highest standard of care for patients in these regions.

## Data Availability

The raw data supporting the conclusions of this article will be made available by the authors, without undue reservation.
