# Supplementary material for: Trends in primary percutaneous coronary intervention for the treatment of acute coronary ST-elevation myocardial infarction in Latin American countries: insights from the CECI consortium
Source: Front Cardiovasc Med. 2024 May 17;11:1275907. doi: 10.3389/fcvm.2024.1275907 (PMC11140057; doi:10.3389/fcvm.2024.1275907)
Supplement: Supplementary file 1 [file Table1.docx]

| **Table S1.** Gender differences, Clinical and Procedural Characteristics and in-hospital outcomes. | | | |
| --- | --- | --- | --- |
|  | Women (n=172) | Men (n=572) | p value |
| Age, years | 70+/- 14 | 62+/- 12 | <0.001 |
| Age>75 years, % | 41.2 | 17.7 | <0.001 |
| Age>90 years, % | 20.0 | 3.9 | <0.001 |
| Symptoms-to-balloon-time, min | 309+/-193 | 290+/-258 | 0.49 |
| Door-to-balloon-time, min (IQR) | 55 (42-69) | 55 (51-60) | 0.98 |
| High blood pressure, % | 75.6 | 53.1 | <0.001 |
| Dislipemia. % | 51.2 | 43.0 | 0.059 |
| Current smoker, % | 18.6 | 25.2 | 0.075 |
| Diabetes, % | 29.1 | 21.7 | 0.045 |
| Family history of CAD, % | 8.1 | 11.2 | 0.25 |
| Known CAD, % | 9.3 | 21.7 | <0.001 |
| Previous stroke, % | 4.7 | 2.1 | 0.067 |
| Previous myocardial infarction, % | 9.3 | 15.4 | 0.044 |
| Previous revascularization procedure, % | 5.8 | 14.7 | 0.002 |
| Procedural Characteristics | | | |
| pPCI, % | 70.9 | 78.0 | 0.057 |
| Femoral access, % | 65.1 | 59.1 | 0.15 |
| Conversion from radial to femoral access, % | 4.7 | 2.1 | 0.067 |
| Left Main as culprit vessel, % | 4.7 | 2.4 | 0.11 |
| Multiple vessel disease, % | 43.0 | 54.2 | 0.010 |
| SCAD, % | 2.3 | 0.0 | 0.003 |
| In-stent thrombosis, % | 3.5 | 8.7 | 0.012 |
| TIMI flow 0 – 1, % | 78.6 | 80.3 | 0.62 |
| Stents per patient | 1.64+/- 1.1 | 1.63 +/- 0.95 | 0.90 |
| Clopidogrel, % | 61.6 | 51.0 | 0.015 |
| No reflow, % | 7.0 | 8.0 | 0.64 |
| Intracoronary IIb/IIIa inhibitors, % | 44.7 | 59.4 | <0.001 |
| Manual thrombectomy,% | 16.3 | 19.1 | 0.40 |
| Contrast, ml | 192+/-72 | 187+/-63 | 0.39 |
| Procedural success, % | 93.0 | 97.2 | 0.012 |
| In-hospital outcomes | | | |
| Cardiogenic Shock, % | 8.1 | 5.2 | 0.112 |
| Intra-aortic balloon pump, % | 0.0 | 2.1 | 0.042 |
| In-hospital death, % | 5.8 | 1.0 | <0.001 |
| CAD: Coronary artery disease; PCI: Percutaneous Coronary Intervention; SCAD: Spontaneous Coronary Artery dissection. | | | |

| **Table S2.** Bleeding complications. | |
| --- | --- |
|  | Overall (%) |
| BARC 3b,4 or 5 | 0,0 |
| BARC 3a | 2,0 |
| Bleeding that required interventions | 1,0 |
| Bleeding that required prolonged hospitalization. | 0,5 |
| Any other bleeding that not fulfill other classifications | 4,9 |
| Overall bleeding | 6,3 |
| BARC 5: Probable or definitive fatal bleeding; BARC 4: bypass related bleeding; BARC 3a: overt bleeding plus a hemoglobin drop of 3 to 5 g/dL* (provided the hemoglobin drop is related to bleed); any transfusion with overt bleeding; BARC 3b: overt bleeding plus a hemoglobin drop of 5 g/dL; BARC 3c: Intracranial hemorrhage. BARC 2: Any overt, actionable sign of hemorrhage that does not fit the criteria for type 3, type 4, or type 5 but does meet at least one of the following criteria: requiring nonsurgical, medical intervention by a health care professional; leading to hospitalization or increased level of care; or prompting evaluation; BARC 1: Beeding that is not actionable. | |
