# Supplementary figures and images for: Trends in primary percutaneous coronary intervention for the treatment of acute coronary ST-elevation myocardial infarction in Latin American countries: insights from the CECI consortium
Source: Front Cardiovasc Med. 2024 May 17;11:1275907. doi: 10.3389/fcvm.2024.1275907 (PMC11140057; doi:10.3389/fcvm.2024.1275907)

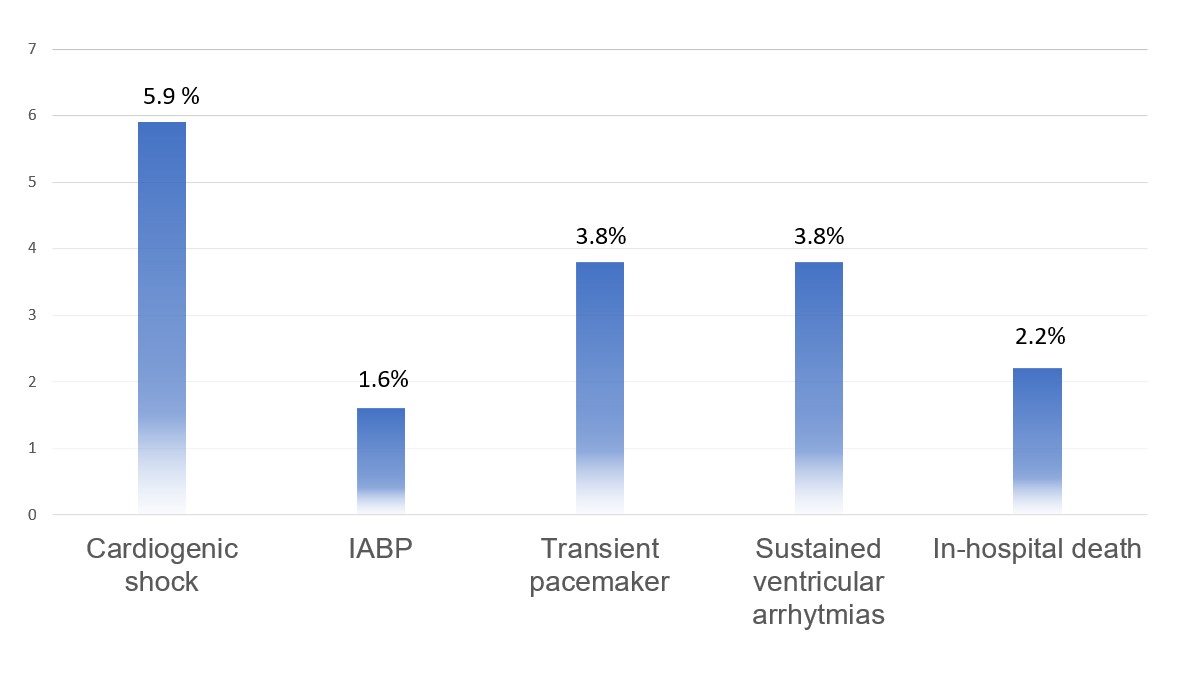

Supplement: Supplementary file 2 [file Image3.jpg]
